# Development and Validation of a Simple Model to Predict the Risk of Nonmelanoma Skin Cancer on Screening Total Body Skin Examination

**DOI:** 10.1155/2022/2313896

**Published:** 2022-08-16

**Authors:** Rebecca I. Hartman, Yun Xue, Ryan Karmouta, Elizabeth Tkachenko, Sara J. Li, David G. Li, Cara Joyce, Arash Mostaghimi

**Affiliations:** ^1^Department of Dermatology, Brigham and Women's Hospital and Harvard Medical School, Boston, MA, USA; ^2^Harvard Combined Dermatology Residency Training Program, Harvard Medical School, Boston, MA, USA; ^3^Cambridge Health Alliance Internal Medicine Residency Training Program, Cambridge, UK; ^4^University of Massachusetts Medical School, Worcester, MA, USA; ^5^Department of Public Health Sciences, Loyola University Stritch School of Medicine, Chicago, IL, USA

## Abstract

**Objective:**

There is insufficient evidence to generate skin cancer screening guidelines at the population level, resulting in arbitrary variation in patient selection for screening skin examinations. This study was aimed at developing an easy-to-use predictive model of nonmelanoma skin cancer (NMSC) risk on screening total body skin examination (TBSE).

**Methods:**

This epidemiologic assessment utilized data from a prospective, multicenter international study from primarily academic outpatient dermatology clinics. Potential predictors of NMSC on screening TBSE were identified and used to generate a multivariable model that was converted into a point-based scoring system. The performance characteristics of the model were validated in a second data set from two healthcare institutions in the United States.

**Results:**

8,501 patients were included. Statistically significant predictors of NMSC on screening TBSE included age, skin phototype, and history of NMSC. A multivariable model and point-based scoring system using these predictors exhibited high discrimination (AUC = 0.82).

**Conclusion:**

A simple three-variable model, abbreviated as CAP (cancer history, age, phototype) can accurately predict the risk of NMSC on screening TBSE by dermatology. This tool may be used in clinical decision making to enhance the yield of screening TBSE.

## 1. Introduction

Nonmelanoma skin cancer (NMSC), including basal and squamous cell carcinoma, is the most common cancer in the U.S. with an estimated 5.4 million cases diagnosed each year [1]. NMSC causes significant morbidity in the U.S. with an annual loss of 230,000 disability-adjusted life years [2]. Treatment delays are associated with tumor growth and may increase morbidity, treatment costs, and patient anxiety [3]. Although screening total body skin examination (TBSE) performed by dermatologists detects skin cancer incidentally [4–8] and may do so earlier than no screening [9–11], there is insufficient evidence to recommend universal screening TBSE by either primary care physicians or dermatologists [12]. Referring providers and dermatologists currently decide on an individual basis that undergoes this intervention, leading to arbitrary practice variation [13, 14].

Current estimates suggest that 41 million U.S. adults (19.8%) have ever undergone screening TBSE [15]. However, only 24.0% of adults at high risk for skin cancer as defined by the United States Preventative Service Task Force have ever had a TBSE, suggesting a discordance between skin cancer risk and screening practices [15]. Systematically identifying patients at high risk for skin cancer may enhance the yield of TBSE screening. Simultaneously, discouraging routine skin cancer screening for patients at low risk may increase dermatology availability for those in need while reducing healthcare costs and unnecessary procedures [16].

To date, there are no simple population-based screening tools to triage patients to screening TBSE by determining an asymptomatic individual's overall risk of NMSC. Two previously published NMSC predictive models each rely on more than 10 features that may be difficult to identify quickly and accurately [17, 18]. We sought to create and validate an easy-to-use, scalable, objective predictive model of NMSC risk to increase the yield of screening TBSE in NMSC detection.

## 2. Methods

### 2.1. Study Setting

The model was generated using primary data from a previously published study by Argenziano et al., which collected data over a time period of 18 months at twelve academic and four private practice dermatology clinics in various South American countries, European countries, and Australia [4] and validated in a cohort from Brigham and Women's Hospital, a large healthcare center in Boston, MA.

### 2.2. Data Collection

Our analysis used data from a previously published prospective, multicenter international study that examined the rates of skin cancer detection via TBSE in patients who presented to dermatology clinics with focused chief complaints [4]. The study began in May 2008 (registration number NCT00765193 at clinicaltrials.gov) and was conducted over a period of 18 months. In that study, dermatologists performed a two-step examination with the aid of dermoscopy as needed, first examining the problem and uncovered areas, and then performing TBSE to detect incidental skin cancers, including melanoma, melanoma in-situ, squamous cell carcinoma, squamous cell carcinoma in situ, and basal cell carcinoma. Participant selection and study design were published previously and excluded patients with chief complaints or primary diagnoses that necessitated TBSE (e.g., diffuse eruption), specific requests for TBSE, and age less than 18 years-old [4]. Skin phototype (SPT), or reaction of the skin to ultraviolet radiation, was ascertained by the dermatologist using the Fitzpatrick method [4].

We additionally excluded patients with signs or symptoms of skin cancer and those with a compelling specific need to go to a dermatologist for TBSE, including patients with a prior history of melanoma, assuming they would receive scheduled TBSE as part of routine follow-up practices. We also excluded patients with chief complaints of lesions of concern (identified by themselves or by referring physicians). The remaining patients lacked lesions of concern and instead had other skin complaints (e.g., acne, skin infections, inflammatory skin diseases, pigmentary abnormalities, and localized rashes) that are not specifically associated with skin cancer risk [4]. We then assessed for NMSC detected by TBSE to identify those detected incidentally by screening. Due to small sample sizes, SPT V and VI were combined in our analysis.

### 2.3. Creation of a Skin Cancer Risk Model

We examined the primary data set for variables associated with the detection of skin cancer on TBSE. These variables were based on previously published and easily identifiable skin cancer risk factors and consisted of the following four variables available from the primary data set: age, gender, SPT, and prior history of NMSC [19]. The history of NMSC was included as a potential strong predictor of future NMSC that could, together with other risk factors, determining the need for follow-up TBSE as not all patients diagnosed with NMSC will necessarily develop subsequent lesions. We used a binary logistic regression model and performed best subsets logistic regression to consider simpler candidate models and formally tested for model improvement using likelihood ratio tests.

Univariable and multivariable logistic regression models were specified using the four independent variables to predict detection of skin cancer on TBSE. Model fit statistics including Akaike's information criterion were used to compare candidate models. We required complete data for inclusion in model building. The final model was converted into points to create a scoring system, where each beta coefficient from the logistic regression model was divided by the lowest beta term (i.e., the lowest log odds ratio) and rounded to the nearest integer [20]. Sensitivity and specificity were calculated for each potential cut-score in the tool. Discrimination of the final model was measured using the area under the receiver operating characteristic (ROC) curve (AUC). Calibration was evaluated with a plot of observed versus predicted risk and assessed formally using the Hosmer–Lemeshow goodness-of-fit test. The chosen cut-score was used to evaluate the tool in our institution's cohort of patients who met similar criteria and who underwent TBSE. SAS 9.4 was used to develop and validate the predictive model (SAS Institute, Cary, NC).

### 2.4. Model Validation

The U.S. validation set for the model came from primary data that we collected from Brigham and Women's Hospital (BWH) from 2010 to 2015, an urban tertiary academic center in Boston, Massachusetts. The Partners Healthcare Institutional Review Board approved this study.

## 3. Results

Of the 14,381 patients enrolled in the original study, 4,954 (34.4%) were excluded due to a chief complaint of skin tumor, 79 (0.5%) were excluded due to a history of melanoma, and 847 (6.9%) were excluded due to missing data (e.g., SPT, history of NMSC, history of melanoma, chief complaint). Of the 8,501 (59.1%) patients who met the inclusion criteria for our analysis, 105 (1.2%) had asymptomatic NMSC detected on screening TBSE [4].

**Figure 1:**
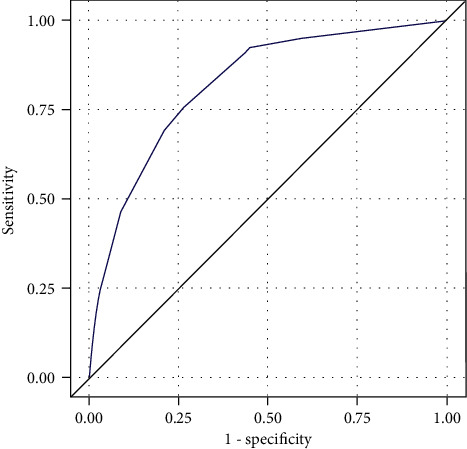
ROC curve for simplified multivariable model. This simplified multivariable model exhibited high discrimination (c-statistic 0.821, 95% CI: 0.783–0.859) using the area under the ROC curve. AUC = 0.821 (95% CI: 0.783–0.859).

**Figure 2:**
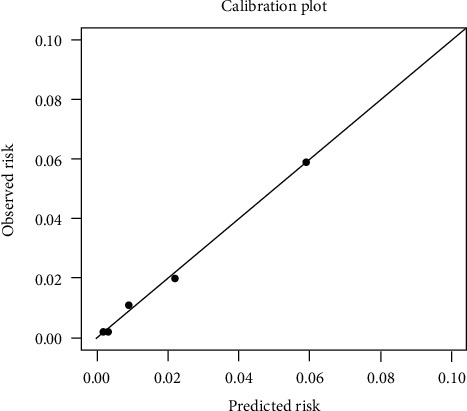
Calibration plot of observed versus predicted risk by quintile. This simplified multivariable model featured good calibration with predicted risk similar to observed risk and using the Hosmer–Lemeshow goodness-of fit test (*χ*_4_^2^ = 1.19, *p*=0.88).

Multivariable analysis identified age, previous history of NMSC, and SPT as significant risk factors for NMSC (c-statistic = 0.831 (95% CI: 0.798–0.865), Table 1). A simplified multivariable model (Table 2) was created using these risk factors, including a previous history of NMSC (RR 3.14, 95% CI 1.85–5.33), age categories as follows: age<50 years-old (RR 1, reference), age 50–64 years-old (RR 5.90, 95% CI: 2.89–12.06), age ≥65 years-old (RR 15.53, 95% CI: 8.05–29.97), and SPT categories as follows: SPT I (RR 3.86, 95% CI 1.87–7.96), SPT II (RR 1.79, 95% CI 1.19–2.69), SPT III-VI (RR 1, reference). This simplified multivariable model exhibited high discrimination (c-statistic 0.821, 95% CI: 0.783–0.859, Figure 1). The model also featured good calibration with predicted risk similar to observed risk (Figure 2) and using the Hosmer–Lemeshow goodness-of fit test (*χ*_4_^2^ = 1.19, *p*=0.88).

We then converted the simplified multivariable model coefficients into a points system (Table 2), which exhibited an ROC curve with similarly high discrimination (c-statistic 0.820, 95% CI: 0.783–0.858). Total point scores range from 0 to 9, with higher scores indicating higher likelihood of detection of NMSC on screening TBSE (Table 3). The Youden Index was optimized with a cut-point of ≥4 at a sensitivity of 77.1% and a specificity of 72.2% (J = 0.49). The implemented cut-point may be modified depending on clinical use and desire to minimize false positives or false negatives (e.g., a score of 3 might be chosen for a test with greater sensitivity at the expense of specificity).

This model was validated using a sample of 1,743 patients from our institution who met inclusion criteria and underwent TBSE by a dermatologist in clinic. A total of 309 in the validation sample did not have skin type available, none of whom had NMSC. Among the remaining 1,434 with complete data on all model predictors and using the above scoring system, 963 (67.2%) scored ≤3 points and 471 (32.8%) scored ≥4 points. There was a significant difference in NMSC detected on TBSE between these two groups (2/963 (0.21%) vs. 9/471 (1.91%), *p* < 0.001). Those with a score ≥4 points were more likely to have NMSC detected on TBSE than those with a score ≤3 points (RR 9.2, 95% CI: 2.0–42.4).

## 4. Discussion

In this study, we present a simple model based on easy-to-determine patient characteristics that identifies asymptomatic patients' overall risk of NMSC on screening TBSE. A previously published predictive model of 3-year NMSC risk exhibited a similar AUC of 0.803 with a sensitivity and specificity of 82.7% and 60.7%, respectively, when Youden's index was optimized. This previously published model relied on 10 self-reported features that may be difficult to identify quickly and accurately (e.g., number of sunburns in past 10 years) [17]. Another previously published predictive model examined 13 parameters using a neural network and yielded a similar AUC of 0.81 and a sensitivity and specificity of 86.2% and 62.7%, respectively, in the validation set [18]. We found that a simple predictive model based on 3 readily discernible factors (age, history of NMSC, and SPT) accurately identifies patients at high risk of having skin cancer on screening TBSE, and we then validated these findings in a U.S. population sample. An electronic medical record could easily identify these features with the exception of SPT, which can be assessed with reliability either from photographs [21] or by a patient self-report [22].

We propose the acronym CAP (cancer history, age, phototype) as a simple mnemonic for this model. At present, use of this predictive model can improve the efficiency of referrals of asymptomatic patients to dermatology for TBSE. When applied at the population level, this model can simultaneously expedite skin cancer diagnosis and treatment by highlighting asymptomatic patients at elevated risk while reducing or postponing lower acuity appointments. Although PCPs can manually use this risk model to identify appropriate referrals to dermatology, a more efficient use would be automated reminders at a population health level.

Dermatologists can use this model to guide their decision to perform screening TBSE on patients with chief complaints other than lesions of concern. 86% of dermatologists report performing screening TBSE at least every 3 years in patients with no personal or family history of skin cancer [14]. Addition of our predictive model may improve the allocation of screening TBSE to those at high risk and enhance the yield of this exam [4]. However, we acknowledge that other factors and features may influence the clinician's decision to perform TBSE, such as immunosuppression, as well as patient preference and request.

Our results must be interpreted in the context of our study design. Our primary dataset was derived from patients referred to dermatology clinics across the world, many of who were academic, and although our model was validated in a U.S. cohort seeking dermatologic care at an academic center, the patient population may not be representative of the U.S. general population [4]. In addition, the training data set was from 2008, and the demographics of these diseases may have shifted over time. That said, validation in an independent data set with data through 2015 suggests that our model is robust and able to be applied to various populations. Our findings were also limited to screening by dermatologists and did not include screening in the primary care setting or by other health professionals. Due to a low event rate, we were unable to evaluate melanoma detection with our dataset. In addition, our study may underestimate the yield of screening TBSE due to Argenizano et al.'s study design, which included uncovered areas, such as the face, in the initial focused examination rather than in the screening TBSE. We acknowledge that our model is simple in design and that the addition of other risk factors may further improve prediction of NMSC development. Nevertheless, our model's simplicity, combined with its high test performance that is similar to previously published more complicated models, may enhance clinical utility in real-world settings.

Although earlier access to care for patients with skin cancer may result in earlier detection, future studies are needed to quantify the degree to which our predictive model can expedite diagnosis and treatment compared to existing clinical care. Furthermore, more data are needed to quantify precisely the potential benefits of earlier skin cancer diagnosis on patient outcomes including morbidity, mortality, and patient anxiety.

Our findings suggest that a simple predictive model can help identify patients who are at high risk of having NMSC on screening TBSE. Implementation of this model for PCP referrals for skin cancer screening and within dermatology clinics may improve triage, leading to earlier NMSC diagnosis and treatment. Further studies are needed to evaluate the implementation of such a scoring system and its effect on patient outcomes.

## Figures and Tables

**Table 1:** Potential predictors of skin cancer on screening TBSE.

	*n*	*n* (%) with skin cancer	Unadjusted odds ratio (95% CI)	Adjusted odds ratio (95% CI)
*Age*				
<35	2580	2 (0.1)	1 (Reference)	1 (Reference)
35–49	2221	8 (0.4)	3.96 (0.97–16.24)	3.84 (0.96–15.43)
50–64	1953	26 (1.3)	14.17 (3.87–51.90)	13.10 (3.64–47.13)
≥65	1747	69 (3.9)	42.68 (12.07–150.95)	33.96 (9.72–118.67)

*Gender*				
Male	3688	55 (1.5)	1.44 (0.98–2.11)	1.28 (0.87–1.89)
Female	4813	50 (1.0)	1 (Reference)	1 (Reference)

*Previous NMSC*				
Yes	248	19 (7.7)	8.02 (4.82–13.35)	3.01 (1.78–5.10)
No	8253	86 (1.0)	1 (Reference)	1 (Reference)

*SPT*				
I	248	9 (3.6)	7.08 (0.89–56.39)	2.57 (0.45–14.76)
II	2556	47 (1.8)	3.52 (0.48–25.67)	1.17 (0.23–6.07)
III	4719	43 (0.9)	1.73 (0.24–12.62)	0.66 (0.13–3.41)
IV	789	5 (0.6)	1.20 (0.14–10.32)	0.54 (0.09–3.31)
V-VI	189	1 (0.5)	1 (Reference)	1 (Reference)

TBSE-total body skin examination, CI-confidence interval; NMSC-nonmelanoma skin cancer; SPT-skin phototype. Exclusion criteria: history of melanoma, symptomatic (e.g., lesion of concern as chief complaint), missing values for above predictors. c-statistic = 0.831 (95% CI: 0.798–0.865) for multivariable model. c-statistic = 0.829 (95% CI: 0.794–0.863) for above multivariable model omitting gender.

**Table 2:** Simplified multivariable model and point scoring system.

	Adjusted odds ratio^*∗*^ (95% CI)	Points^∗∗^
*Age*		
<50	1 (Reference)	0
50–64	5.90 (2.89–12.06)	3
≥65	15.53 (8.05–29.97)	5

*Previous NMSC*		
Yes	3.14 (1.85–5.33)	2
No	1 (Reference)	0

*SPT*		
I	3.86 (1.87–7.96)	2
II	1.79 (1.19–2.69)	1
III-VI	1 (Reference)	0

CI-confidence interval; NMSC-nonmelanoma skin cancer, SPT-skin phototype. ^*∗*^c-statistic = 0.821 (95% CI: 0.783–0.859); Hosmer–Lemeshow goodness of fit test *p*=0.88 for simplified multivariable model. ^∗∗^maximum of 9 points possible (age ≥ 65, NMSC, SPT I).

**Table 3:** Classification statistics for cut points in the TBSE model.

	*n* positive	Sensitivity	Specificity
*Points*			
≥2	3862	92.4%	55.2%
≥3	3718	90.5%	56.8%
≥4^*∗*^	2419	77.1%	72.2%
≥5	1858	69.5%	78.7%
≥6	758	44.8%	91.5%
≥7	216	21.0%	97.7%
≥8	83	10.5%	99.1%

TBSE-total body skin examination. ^*∗*^4 is the optimal cut point by Youden's index.

## Data Availability

The model was generated using primary data from a previously published study by Argenziano et al., which collected data over a time period of 18 months at twelve academic and four private practice dermatology clinics in various South American countries, European countries, and Australia.
